# Prevalence and characteristics of methicillin-resistant coagulase-negative staphylococci from livestock, chicken carcasses, bulk tank milk, minced meat, and contact persons

**DOI:** 10.1186/1746-6148-7-6

**Published:** 2011-01-27

**Authors:** Helen Huber, Dominik Ziegler, Valentin Pflüger, Guido Vogel, Claudio Zweifel, Roger Stephan

**Affiliations:** 1 Institute for Food Safety and Hygiene, Vetsuisse Faculty University of Zurich, Winterthurerstrasse 272, 8057 Zurich, Switzerland; 2 Mabritec AG, Lörracherstrasse 50, Postfach 320, 4125 Riehen BS, Switzerland

## Abstract

**Background:**

Methicillin-resistant coagulase-negative staphylococci (MR-CNS) are of increasing importance to animal and public health. In veterinary medicine and along the meat and milk production line, only limited data were so far available on MR-CNS characteristics. The aim of the present study was to evaluate the prevalence of MR-CNS, to identify the detected staphylococci to species level, and to assess the antibiotic resistance profiles of isolated MR-CNS strains.

**Results:**

After two-step enrichment and growth on chromogenic agar, MR-CNS were detected in 48.2% of samples from livestock and chicken carcasses, 46.4% of samples from bulk tank milk and minced meat, and 49.3% of human samples. Using matrix-assisted laser desorption ionization-time of flight mass spectrometry (MALDI-TOF MS), 414 selected MR-CNS strains belonged to seven different species (*S. sciuri*, 32.6%; *S. fleurettii*, 25.1%; *S. haemolyticus*, 17.4%; *S. epidermidis*, 14.5%, *S. lentus*, 9.2%; *S. warneri*, 0.7%; *S. cohnii*, 0.5%). *S. sciuri *and *S. fleurettii *thereby predominated in livestock, BTM and minced meat samples, whereas *S. epidermidis *and *S. haemolyticus *predominated in human samples. In addition to beta-lactam resistance, 33-49% of all 414 strains were resistant to certain non-beta-lactam antibiotics (ciproflaxacin, clindamycin, erythromycin, tetracycline).

**Conclusions:**

A high prevalence of MR-CNS was found in livestock production. This is of concern in view of potential spread of *mec*A to *S. aureus *(MRSA). Multiresistant CNS strains might become an emerging problem for veterinary medicine. For species identification of MR-CNS isolated from different origins, MALDI-TOF MS proved to be a fast and reliable tool and is suitable for screening of large sample amounts.

## Background

It is assumed that methicillin-resistance genes had evolved in coagulase-negative staphylococci (CNS) and were then horizontally transferred among staphylococci [1,2]. Particularly *S. sciuri *[3] and *S. fleurettii *[4] are discussed as natural reservoir of the methicillin-resistance gene *mec*A. The *mec*A gene is located on a mobile genetic element called staphylococcal cassette chromosome (SCC) and confers resistance to methicillin by encoding an altered penicillin-binding protein (PBP2α), which shows limited affinity to beta-lactam antibiotics. The *mec*A gene is now distributed among both coagulase-positive and -negative staphylococcal species.

In recent years, the isolation of methicillin-resistant CNS (MR-CNS) from diverse sources was reported. Dakic et al. [5] detected MR-CNS on medical devices, Silva et al. [6] in healthy humans, de Mattos et al. [7] and Ruppe et al. [8] in ambulatory patients, and Diekema et al. [9] in human bloodstream infections. According to Kloos and Bannerman [10], *S. epidermidis *and *S. haemolyticus *are the principal pathogens involved in foreign body infections. Miragaia et al. [11] discussed *S. epidermidis *as one of the most important causes for hospital-acquired bacteremia and found many isolates from hospitalized patients to be methicillin-resistant. Recently, several authors reported MR-CNS also in healthy animals. The existence of MR-CNS in animals was first described by Kawano et al. [12], who detected MR-CNS in chicken. Worldwide, MR-CNS were isolated from horses [13-15], dogs [15], cattle [16,17], as well as from sheep, goats, and pigs [17]. However, MR-CNS were also found in animals with clinical infections. Van Duijkeren et al. [18] detected methicillin-resistant *S. haemolyticus *from cats with cystitis and rhinitis, from dogs with bronchitis and pyoderma, and from a horse with vaginitis. Moreover, Fessler et al. [19] reported different MR-CNS species in association with bovine mastitis.

However, comprehensive data on MR-CNS along the meat and milk production line were so far missing. The present work is a subsequent study of a previously published study on the prevalence of MRSA in Switzerland. The aim of the present study was to evaluate the occurrence of MR-CNS in livestock (cows, calves, pigs) and chicken carcasses, bulk tank milk (BTM) and minced meat, as well as people in contact with farm animals (veterinarians, pig farmers, and employees of a chicken and a pig and cattle abattoir), to identify the occurring CNS species, and to assess antibiotic resistance profiles of isolated strains.

## Results

### MR-CNS occurrence

Using the described procedure, MR-CNS were detected in 1'013 (48.3%) of the 2'099 analyzed samples from livestock and chicken carcasses, BTM and minced meat, and humans (Table 1). Amongst the 1'428 samples from livestock and chicken carcasses, MR-CNS were found in 48.2% of samples. Thereby, the proportion of positive samples ranged from 36.3% in pigs to 72.0% in calves. Analysis of the 211 samples from BTM and minced meat yielded an average MR-CNS occurrence of 46.4% (32.4% in meat and 62.0% in BTM). Of the 460 human samples, 49.3% turned out to test positive for MR-CNS (from 26.3% in slaughterhouse employees to 67.6% in pig farmers).

**Table 1:** Prevalence of MR-CNS strains detected in samples from livestock, chicken carcasses, bulk tank milk, minced meat, and contact persons.

Origin	No. of sampled animals, humans and products	No. of MR-CNS positive samples (%)	No. of MR-CNS isolates
Pigs (from 241 farms)^1)^	716^3)^	260 (36.3)	260
Cows (from 253 farms)^1)^	340^4)^	177 (52.1)	180
Calves (from 187 farms)^1)^	300^4)^	216 (72.0)	218
Chicken (from 72 farms)^2)^	72	35 (48.6)	38
Bulk tank milk (from 100 farms)	100	62 (62.0)	62
Minced meat	111	36 (32.4)	36
Veterinarians^1)^	133	80 (60.2)	84
Pig farmers^1)^	148	100 (67.6)	105
Slaughterhouse employees^1)^	179	47 (26.3)	48
Total	2099	1013 (48.3)	1031

1) Nasal swabs.

2) Herd-wise pooled neck skin samples.

3) At most three animals per farm were sampled.

4) At most two animals per farm were sampled.

### Species identification

A total of 414 strains were selected and subjected to species identification using MALDI-TOF MS. Amongst samples, seven different CNS species were detected (Table 2). Sequencing of *sod*A from a subset of 25 strains (5 *S. sciuri*; 5 *S. fleurettii; *5 *S. haemolyticus; *5 *S. epidermidis; *3 *S. lentus; *1 *S. cohnii; *1 *S. warneri*) confirmed the results obtained by MALDI-TOF MS. Overall, *S. sciuri *(32.6%) and *S. fleurettii *(25.1%) were most frequently found, followed by *S. haemolyticus *(17.4%), *S. epidermidis *(14.5%), and *S. lentus *(9.2%). Moreover, *S. cohnii *and *S. warneri *were only rarely identified (<1.0%).

**Table 2:** Species distribution of MR-CNS strains from different origins.

		Species distribution						
		
Origin	No. of strains	*S. sciuri*	*S. fleurettii*	*S. haemolyticus*	*S. epidermidis*	*S. lentus*	*S. warneri*	*S. cohnii*
Pigs	52	29	23	0	0	0	0	0
Cows	50	36	11	2	0	0	1	0
Calves	51	50	0	1	0	0	0	0
Poultry	38	6	0	0	2	30	0	0
BTM	50	11	37	1	0	0	0	1
Minced meat	34	2	27	2	2	0	1	0
Veterinarians	50	0	1	33	14	0	1	1
Pig farmers	50	0	2	23	25	0	0	0
SH	39	1	3	10	17	8	0	0
Total	414	135	104	72	60	38	3	2

BTM: bulk tank milk; SH: Slaughterhouse employees

Within the 191 selected MR-CNS strains of animal origin, the majority of strains was identified as *S. sciuri *(63.4% of strains), followed by *S. fleurettii *(17.8%), *S. lentus *(15.7%), *S. haemolyticus *(1.6%), *S. epidermidis *(1.0%), and *S. warneri *(0.5%). Thirty of the 38 *S. lentus *strains isolated originated from chicken. Of the 51 strains isolated from calves, 50 were identified as *S. sciuri*. The 52 strains from pigs were identified as *S. sciuri *(55.8%) and *S. fleurettii *(44.2%). The 50 MR-CNS strains isolated from cows also mainly consisted of *S. sciuri *(72.0%) and *S. fleurettii *(22.0%).

Of the 84 MR-CNS strains isolated from BTM and minced meat samples, 76.2% of strains were identified as *S. fleurettii*, followed by 15.5% of strains identified as *S. sciuri*. The remaining seven strains belonged to *S. haemolyticus *(n = 3), *S. epidermidis *(n = 2), *S. cohnii *(n = 1), and *S. warneri *(n = 1).

Of the 139 MR-CNS strains isolated from humans, *S. haemolyticus *(47.5%) and *S. epidermidis *(40.3%) accounted for the great majority. *S. lentus *were only isolated from eight employees of a poultry slaughterhouse, whereas *S. fleurettii *was found in a veterinarian, two pig farmers, and three slaughterhouse employees. The remaining three MR-CNS strains isolated from two veterinarians and a slaughterhouse employee were identified as *S. cohnii*, *S. warneri *and *S. sciuri*.

### Antimicrobial susceptibility testing

The results of the phenotypic antibiotic resistance testing of the 414 MR-CNS strains are shown in Tables 3, 4 and 5. Although all characterized strains harbored the *mec*A gene, phenotypic resistance to beta-lactam antibiotics (ampicillin, cefoxitin, oxacillin, penicillin) varied between 63.5% (cefoxitin) and 97.8% (oxacillin). Resistance to ciprofloxacin, clindamycin, erythromycin, gentamicin, sulphamethoxazole/trimethoprim, and tetracycline varied between 14.7% and 49.0%. On the other hand, resistance to rifampin and vancomycin was only detected in 0.7% and 0.2% of strains, respectively. Table 5 shows the species-specific situation of MR-CNS strains to cefoxitin.

**Table 3:** Species distribution and antibiotic resistance of the 414 MR-CNS strains.

		Percentage of resistant strains											
		
Species	No. of strains	Oxa	Pen	Amp	Cef	Ery	Tet	Cli	Cip	SxT	Gen	Rif	Van
*S. sciuri*	135	100	97.8	97.8	83.7	66.7	77.8	65.2	40.7	11.9	19.3	2.2	0
*S. fleurettii*	104	100	65.4	66.3	32.7	5.8	4.8	18.3	0	0	0	0	1.0
*S. haemolyticus*	72	90.3	95.8	95.8	87.5	88.9	80.6	65.3	80.6	72.2	31.9	0	0
*S. epidermidis*	60	96.7	86.7	75.0	46.7	58.3	48.3	25.0	35.0	13.3	20.0	0	0
*S. lentus*	38	100	78.9	86.8	52.6	18.4	15.8	28.9	2.6	5.3	0	0	0
*S. warneri*	3	100	100	100	100	0	0	0	0	0	0	0	0
*S. cohnii*	2	100	100	100	100	50.0	0	50.0	0	0	0	0	0
Total	414	97.8	86.0	85.3	63.5	49.0	49.0	43.7	32.6	18.8	14.7	0.7	0.2

Amp: ampicillin; Cef: cefoxitin; Cip: ciprofloxacin; Cli: clindamycin; Ery: erythromycin; Gen: gentamicin; Oxa: oxacillin; Pen: penicillin; Rif: rifampin; SxT: sulphamethoxazole/trimethoprim; Tet: tetracycline;Van: vancomycin.

**Table 4:** Origin and antibiotic resistance of the 414 MR-CNS strains.

		Percentage of resistant strains											
		
Origin	No. of strains	Oxa	Pen	Amp	Cef	Ery	Tet	Cli	Cip	SxT	Gen	Rif	Van
Pigs	52	100	75.0	76.9	53.8	26.9	44.2	25.0	3.8	15.4	19.2	0	1.9
Cows	50	100	94.0	96.0	90.0	54.0	58.0	58.0	44.0	4.0	8.0	4.0	0
Calves	51	100	96.1	96.1	70.6	88.2	92.2	86.3	62.7	15.7	17.6	0	0
Chicken	38	100	78.9	86.8	68.4	18.4	18.4	18.4	5.3	2.6	2.6	0	0
Bulk tank milk	50	100	76.0	78.0	42.0	10.0	8.0	4.0	6.0	0	2.0	0	0
Minced meat	34	100	73.5	70.6	35.3	8.8	11.8	5.9	8.8	0	2.9	0	0
Veterinarians	50	96.0	94.0	90.0	78.0	80.0	84.0	58.0	64.0	64.0	26.0	0	0
Pig farmers	50	96.0	100	98.0	74.0	72.0	76.0	56.0	62.0	40.0	38.0	0	0
Sh employees	39	87.2	74.4	66.7	38.5	48.7	12.8	23.1	25.6	12.8	5.1	2.6	0
Total	414	97.7	84.7	84.3	61.2	45.2	45.0	37.2	31.4	17.2	13.5	0.7	0.2

Amp: ampicillin; Cef: cefoxitin; Cip: ciprofloxacin; Cli: clindamycin; Ery: erythromycin; Gen: gentamicin; Oxa: oxacillin; Pen: penicillin; Rif: rifampin; Sh: slaughterhouse; SxT: sulphamethoxazole/trimethoprim; Tet: tetracycline; Van: vancomycin.

**Table 5:** Resistance to cefoxitin: distribution of the number of strains in each staphylococcal species with regard to mm inhibition zone (bold: resistant strains).

mm inhibition zone	6	11	12	13	14	15	16	17	18	19	20	21	22	23	24	25	26	27	28	29	30	31	32	33	34	35	36
*S. cohnii*							**1**		**1**																		
*S. epidermidis*	**1**		**2**		**1**		**3**		**4**		**3**		**6**	**3**	**5**	4	10	3	4		2	1	1	1	3		3
*S. fleurettii*						**2**	**1**			**1**	**4**	**5**	**11**	**5**	**4**	3	8	1	12	1	10	2	15	2	10		6
*S. haemolyticus*	**2**	**2**	**1**	**3**	**1**	**6**	**2**	**6**	**5**	**6**	**8**	**2**	**9**	**3**	**7**	1	3		3		1		1				
*S. lentus*								**1**	**2**	**3**	**2**	**4**	**3**	**1**	**4**	1	3	2	4		3		3		2		
*S. sciuri*	**2**	**1**	**1**	**4**	**5**	**4**	**6**	**4**	**9**	**4**	**8**	**11**	**21**	**19**	**14**	10	9	2		1							
*S. warneri*		**2**	**1**																								

With regard to resistance rates of individual MR-CNS species (Table 3), more than 87% of the *S. sciuri *and *S. haemolyticus *strains showed phenotypical resistance to beta-lactam antibiotics. On the other hand, of the *S. epidermidis*, *S. fleurettii*, and *S. lentus *strains only 60% to 80% were resistant to beta-lactam antibiotics. Apart from one exception, more than 54% of strains from cows, calves, veterinarians and pig farmers were resistant to ciprofloxacin, clindamycin, erythromycin, and tetracycline (Table 4). In contrast, apart from two exceptions, less than 27% of strains isolated from BTM, minced meat, pigs and slaughterhouse employees, were resistant to the four antibiotics mentioned above. No strains from BTM, minced meat, and chicken were resistant to sulphamethoxazole/trimethoprim (SxT), whereas 40.0% of strains from pig farmers and 64.0% of strains from veterinarians showed resistance to SxT. None of the strains isolated from BTM and minced meat samples were resistant to rifampin or vancomycin.

## Discussion

Methicillin-resistant staphylococci are a major concern to public and animal health. In veterinary medicine and along the meat and milk production line only very limited data were available on characteristics of MR-CNS. The present study provides an overview on the MR-CNS situation in Swiss livestock, chicken carcasses, BTM, minced meat, and persons in contact with farm animals. Since data of studies with comparable procedures are lacking, direct comparison of results is not possible. Therefore, we discussed our results in comparison to data that we considered most appropriate and as close to our own results as possible. In our study, MR-CNS were detected at an average of 48.3% in 2'099 samples. The occurrence varied thereby from 26.3% in slaughterhouse employees to 72.0% in calves. The MR-CNS occurrence rates mentioned above are in contrast to the low MRSA prevalence in Switzerland [20].

In the present study, 48.2% of all samples from livestock (pigs, cows, calves) and chicken carcasses tested positive for MR-CNS. MR-CNS were also found in cows and pigs by Zhang et al. [17]. In contrast, Bagcigil et al. [15] did not detect any MR-CNS in pigs and cattle. The species most frequently detected in animal samples of the present study were *S. sciuri *(63.4%), followed by *S. fleurettii *(17.8%), and *S. lentus *(15.7%). In calves, 98.0% of MR-CNS strains were identified as *S. sciuri*, whereas in cows only 72.0% belonged to this species. The strains from pigs were divided about half-and-half in *S. sciuri *and *S. fleurettii*. The high percentage of *S. sciuri *in pigs is especially relevant, since this CNS species is also reported to cause fatal exudative epidermitis in piglets [21], which is normally caused by exfoliative toxin producing *S. hyicus*. Interestingly, methicillin-resistant *S. sciuri *were also found to predominate in horses [22,23]. Furthermore, the MR-CNS strains isolated from chicken in our study were mainly (78.9%) of *S. lentus*, whereas Kawano et al. [12] did not detect methicillin-resistant *S. lentus *in their samples. Methicillin-resistant *S. lentus *have also been described in other animal species such as horses [14,22,24], pigs, cattle, sheep, and goats [17]. Other data on species-specific identification of MR-CNS from livestock are limited.

In the BTM and minced meat samples of the present study, MR-CNS were detected in 32.4% of minced meat and in 62.0% of BTM samples. Respective data on MR-CNS from these sample categories are hardly found. The species predominantly found in BTM and minced meat samples of the present study, were *S. fleurettii *accounting for 76.2% of all analyzed strains, followed by *S. sciuri *comprising 15.5% of strains. In bovine quarter milk samples, Gillespie et al. [25] found mainly CNS of *S. chromogenes*, *S. hyicus*, *S. epidermidis*, and *S. simulans*. In a subsequent study, Sawant et al. [26] found 11 of 37 *S. epidermidis *strains to harbor the *mec*A gene and show phenotypic resistance to ampicillin and oxacillin. On the other hand, CNS are also frequently isolated from clinical and in particular subclinical intramammary infections in cattle [27] and small ruminants [28-30]. CNS found in this context generally showed a species distribution different from that present in our samples.

In the 460 nasal swabs from humans in contact with farm animals, MR-CNS were detected in 49.3% of samples. In view of contact persons, two studies found the prevalence of MR-CNS in horse personnel to be 37% and 63% [22,23]. Moreover, a study among healthy humans without association to hospital or animals found an MR-CNS prevalence of 24% [6]. In human samples of the present study, the most frequently isolated species were *S. haemolyticus *(47.5%) and *S. epidermidis *(40.3%). MR-CNS of species *S. haemolyticus *[23] and *S. epidermidis *[22] were also frequently isolated from horse personnel. These two predominant species are also of importance for human infections. As discussed above, *S. epidermidis *and *S. haemolyticus *are frequently isolated CNS species involved in clinical cases of humans.

There are not much data available on the antimicrobial susceptibility of MR-CNS isolated from colonized livestock and humans, or from BTM and minced meat. In the present study, MR-CNS strains from BTM, minced meat, and chicken were only in limited numbers resistant to antibiotics other than beta-lactams. In view of MR-CNS as food contaminants and potential vectors of antibiotic resistance, these results suggest a favorable situation. In contrast, strains isolated from calves, cows, pig farmers, and veterinarians showed multi-resistance to a great extent. This situation may also be caused by frequent direct contact of these individuals with antibiotics. Interestingly, resistance rates of strains from pigs and slaughterhouse employees were in-between the groups mentioned above. Furthermore, it is noteworthy that in our MR-CNS strains, phenotypic resistance to oxacillin was by far a better indicator for the presence of the *mec*A gene than resistance to cefoxitin. Interestingly, for indication of *mec*A in MR-CNS using MIC, both cefoxitin and oxacillin are discussed as valuable approaches [31].

## Conclusion

In conclusion, the results obtained indicate a high MR-CNS prevalence (on average 48.3%) in samples from livestock, chicken carcasses, BTM and minced meat, as well as persons in contact with livestock. The occurrence thereby ranged from 26.3% in slaughterhouse employees to 72.0% in calves. This is the first study detecting MR-CNS in a large collection of samples taken along the meat and milk production line. For species identification of MR-CNS isolated from different origins, MALDI TOF MS proved to be a fast and reliable tool and is therefore suitable for screening of large sample amounts. Furthermore, based on the present data, the occurrence of methicillin-resistant staphylococci should be continuously evaluated in surveillance programs within the scope of veterinary public health aspects. Otherwise, further studies are required to investigate the impact of the high MR-CNS prevalence on human and animal health.

## Methods

### Bacterial strains

From March through September 2009, we collected a total of 2'099 samples from livestock and chicken carcasses, BTM and minced meat, as well as humans in contact with farm animals. Livestock was sampled at slaughter. Thereby, swabs were collected from the nasal cavity of pigs (n = 716), calves (n = 300), cows (n = 340). From chicken carcasses herd-wise pooled neck skin samples (n = 72) were collected, each herd originating from a different farm. Sampled animals (including chicken) originated from more than 800 farms in 10 different cantons distributed throughout Switzerland. Furthermore, 100 BTM and 111 minced meat (pork and beef) samples were tested. Moreover, we analyzed nasal swabs from 148 pig farmers attending two meetings on swine breeding, 133 veterinarians participating in a course on castration of piglets, and 179 slaughterhouse employees working in two different abattoirs (a poultry and a pig and cattle slaughterhouse). All human nasal swabs samples were taken by the persons themselves. They gave us their consent that the samples can be used for this study.

For isolation of MR-CNS strains, a two-step enrichment procedure in Mueller-Hinton broth supplemented with 6.5% NaCl (24 h at 37°C) and in phenolred mannitol broth supplemented with 75 mg/L aztreonam and 5 mg/L cefoxitin (24 h at 37°C) was used. Samples were then plated onto Oxoid Brilliance™ MRSA Agar (Oxoid Ltd., Hampshire, UK) and incubated for 24 h at 37°C. Of presumptive MR-CNS colonies of beige or white color, one colony (and for some samples with different colony morphologies at most two colonies) was picked, gram-stained, and tested for catalase-activity. All gram-positive, catalase-positive cocci grown beige or white on Oxoid Brilliance™ MRSA Agar were considered MR-CNS. Thereafter, a random selection of 414 strains (34 to 52 isolates per sample origin) was confirmed as genetically methicillin-resistant by detection of the *mec*A gene [32] and then further characterized.

### Species identification

MR-CNS species identification was performed by matrix-assisted laser desorption ionization-time of flight mass spectrometry (MALDI-TOF MS). MALDI-TOF MS was recently described as an effective and powerful tool for reliable identification of staphylococci [33] and CNS in particular [34]. Briefly, of the 414 randomly selected strains, one or two colonies of a fresh overnight culture grown on TSA were suspended in 20 μL formic acid (25%). Thereafter, 20 μL of a saturated solution of sinapinic acid (49508, Sigma-Aldrich, Buchs, Switzerland) in 60% acetonitril (154601, Sigma-Aldrich, Buchs, Switzerland), 0.3% trifluoracetic acid (T6508, Sigma-Aldrich, Buchs, Switzerland) was added. Of this mixture 1 μl was spotted in duplicates on a steel target plate and air dried at room temperature. Spectra were acquired using a Axima™ confidence (Shimadzu-Biotech Corp., Kyoto, Japan) MALDI-TOF massspectrometer, in the linear positive mode, at a laser frequency of 50 Hz and within a mass range from 2000-30000 Da. Acceleration voltage was 20 kV, and the extraction delay time was 200 ns. A minimum of 10 lasershots per sample was used to generate each ion spectrum. For each bacterial sample, a total of 100 ion spectra were averaged and processed using the Launchpad™ v.2.8 Software (Shimadzu-Biotech Corp., Kyoto, Japan). Peaks lists were in ASCII format imported into the SARAMIS™ (Spectral Archive And Microbial Identification System, AnagnosTec, Potsdam-Golm, Germany) for automated species identification.

For additional confirmation of species identification, 56 randomly chosen strains were identified by sequencing an internal fragment of the *sod*A gene following a protocol of Poyart et al. [35] with slight modifications. DNA sequence-based species identification is currently the most accurate identification method available for CNS [36]. The partial *sod*A gene was amplified by PCR using primers d1 (5'-CCITAYICITAYGAYGCIYTIGARCC-3') and d2 (5'-ARRTARTAIGCRTGYTCCCAIACRTC-3'). PCRs were performed in a T3000 thermocycler (Biometra, Goettingen, Germany) in a final volume of 50 μL containing a minimum of 150 ng of DNA, 2 μM of each primer, 200 μM of each deoxynucleoside triphosphate, and 2 U of FastStart High Fidelity polymerase (Roche, Basel, Switzerland) in a 10× reaction buffer (with 18 mM MgCl_2_, included in kit). PCR mixtures were denatured (3 min, 95°C) and then subjected to 30 cycles of amplification (60 s of annealing at 39°C, 45 s of elongation at 72°C, and 30 s of denaturation at 95°C). The product was then sequenced and the results were compared to the GenBank database using the BLAST program (http://blast.ncbi.nlm.nih.gov/Blast.cgi).

### Antimicrobial susceptibility testing

To evaluate the phenotypic antibiotic resistance of the selected 414 strains, the disk diffusion method was used in accordance with the guidelines of the Clinical and Laboratory Standards Institute [37] using the following disks (BD BBL Sensi-Disc; Becton, Dickinson and Company, Sparks, MD, USA): ampicillin (10 μg), cefoxitin (30 μg), ciprofloxacin (5 μg), clindamycin (2 μg), erythromycin (15 μg), gentamicin (10 μg), oxacillin (1 μg), penicillin (10 units), rifampin (5 μg), sulphamethoxazole/trimethoprim (23.75/1.25 μg), tetracycline (30 μg), and vancomycin (30 μg). Results were interpreted according to the CLSI guidelines, whereby intermediate results were considered resistant.

## Authors' contributions

HH was responsible for isolation and characterization of strains and drafted the manuscript. DZ, VP, and GV performed MALDI-TOF MS analysis and evaluated respective data. CZ and RS designed the study and edited the manuscript. All authors read, commented on, and approved the final manuscript.

## References

[B1] Barbier F Ruppe E Hernandez D Lebeaux D Francois P Felix B Desprez A Maiga A Woerther PL Gaillard K Methicillin-resistant coagulase-negative staphylococci in the community: high homology of SCCmec IVa between *Staphylococcus epidermidis *and major clones of methicillin-resistant *Staphylococcus aureus* J Infect Dis 2010 202 270 281 10.1086/653483 20550456

[B2] Archer GL Niemeyer DM Thanassi JA Pucci MJ Dissemination among staphylococci of DNA sequences associated with methicillin resistance Antimicrob Agents Chemother 1994 38 447 454 7911288 10.1128/aac.38.3.447 PMC284478

[B3] Kloos WE Ballard DN Webster JA Hubner RJ Tomasz A Couto I Sloan GL Dehart HP Fiedler F Schubert K Ribotype delineation and description of *Staphylococcus sciuri *subspecies and their potential as reservoirs of methicillin resistance and staphylolytic enzyme genes Int J Syst Bacteriol 1997 47 313 323 10.1099/00207713-47-2-313 9103615

[B4] Tsubakishita S Kuwahara-Arai K Sasaki T Hiramatsu K Origin and molecular evolution of the determinant of methicillin resistance in staphylococci Antimicrob Agents Chemother 2010 54 4352 4359 10.1128/AAC.00356-10 20679504 PMC2944575

[B5] Dakic I Morrison D Vukovic D Savic B Shittu A Jezek P Hauschild T Stepanovic S Isolation and molecular characterization of *Staphylococcus sciuri *in the hospital environment J Clin Microbiol 2005 43 2782 2785 10.1128/JCM.43.6.2782-2785.2005 15956397 PMC1151920

[B6] Silva FR Mattos EM Coimbra MV Ferreira-Carvalho BT Figueiredo AM Isolation and molecular characterization of methicillin-resistant coagulase-negative staphylococci from nasal flora of healthy humans at three community institutions in Rio de Janeiro City Epidemiol Infect 2001 127 57 62 10.1017/S095026880100574X 11561975 PMC2869729

[B7] de Mattos EM Teixeira LA Alves VM Rezendae Resende CA da Silva Coimbra MV da Silva-Carvalho MC Ferreira-Carvalho BT Figueiredo AM Isolation of methicillin-resistant coagulase-negative staphylococci from patients undergoing continuous ambulatory peritoneal dialysis (CAPD) and comparison of different molecular techniques for discriminating isolates of *Staphylococcus epidermidis* Diagn Microbiol Infect Dis 2003 45 13 22 10.1016/S0732-8893(02)00477-7 12573546

[B8] Ruppe E Barbier F Mesli Y Maiga A Cojocaru R Benkhalfat M Benchouk S Hassaine H Maiga I Diallo A Diversity of staphylococcal cassette chromosome mec structures in methicillin-resistant *Staphylococcus epidermidis *and *Staphylococcus haemolyticus *strains among outpatients from four countries Antimicrob Agents Chemother 2009 53 442 449 10.1128/AAC.00724-08 19001111 PMC2630651

[B9] Diekema DJ Pfaller MA Schmitz FJ Smayevsky J Bell J Jones RN Beach M Survey of infections due to *Staphylococcus *species: frequency of occurrence and antimicrobial susceptibility of isolates collected in the United States, Canada, Latin America, Europe, and the Western Pacific region for the SENTRY Antimicrobial Surveillance Program, 1997-1999 Clin Infect Dis 2001 32 Suppl 2 S114 132 10.1086/320184 11320452

[B10] Kloos WE Bannerman TL Update on clinical significance of coagulase-negative staphylococci Clin Microbiol Rev 1994 7 117 140 8118787 10.1128/cmr.7.1.117 PMC358308

[B11] Miragaia M Couto I Pereira SF Kristinsson KG Westh H Jarlov JO Carrico J Almeida J Santos-Sanches I de Lencastre H Molecular characterization of methicillin-resistant *Staphylococcus epidermidis *clones: evidence of geographic dissemination J Clin Microbiol 2002 40 430 438 10.1128/JCM.40.2.430-438.2002 11825953 PMC153385

[B12] Kawano J Shimizu A Saitoh Y Yagi M Saito T Okamoto R Isolation of methicillin-resistant coagulase-negative staphylococci from chickens J Clin Microbiol 1996 34 2072 2077 8862560 10.1128/jcm.34.9.2072-2077.1996 PMC229192

[B13] Yasuda R Kawano J Matsuo E Masuda T Shimizu A Anzai T Hashikura S Distribution of *mecA*-harboring staphylococci in healthy mares J Vet Med Sci 2002 64 821 827 10.1292/jvms.64.821 12399608

[B14] De Martino L Lucido M Mallardo K Facello B Mallardo M Iovane G Pagnini U Tufano MA Catalanotti P Methicillin-resistant staphylococci isolated from healthy horses and horse personnel in Italy J Vet Diagn Invest 2010 22 77 82 20093688 10.1177/104063871002200114

[B15] Bagcigil FA Moodley A Baptiste KE Jensen VF Guardabassi L Occurrence, species distribution, antimicrobial resistance and clonality of methicillin- and erythromycin-resistant staphylococci in the nasal cavity of domestic animals Vet Microbiol 2007 121 307 315 10.1016/j.vetmic.2006.12.007 17270365

[B16] Jaglic Z Michu E Holasova M Vlkova H Babak V Kolar M Bardon J Schlegelova J Epidemiology and characterization of *Staphylococcus epidermidis *isolates from humans, raw bovine milk and a dairy plant Epidemiol Infect 2010 138 772 782 10.1017/S0950268809991002 19845994

[B17] Zhang Y Agidi S LeJeune JT Diversity of staphylococcal cassette chromosome in coagulase-negative staphylococci from animal sources J Appl Microbiol 2009 107 1375 1383 10.1111/j.1365-2672.2009.04322.x 19486384

[B18] van Duijkeren E Box AT Heck ME Wannet WJ Fluit AC Methicillin-resistant staphylococci isolated from animals Vet Microbiol 2004 103 91 97 10.1016/j.vetmic.2004.07.014 15381271

[B19] Fessler AT Billerbeck C Kadlec K Schwarz S Identification and characterization of methicillin-resistant coagulase-negative staphylococci from bovine mastitis J Antimicrob Chemother 2010 65 1576 1582 10.1093/jac/dkq172 20525989

[B20] Huber H Koller S Giezendanner N Stephan R Zweifel C Prevalence and characteristics of meticillin-resistant *Staphylococcus aureus *in humans in contact with farm animals, in livestock, and in food of animal origin, Switzerland, 2009 Euro Surveill 2010 15 20430001

[B21] Chen S Wang Y Chen F Yang H Gan M Zheng SJ A highly pathogenic strain of *Staphylococcus sciuri *caused fatal exudative epidermitis in piglets PLoS One 2007 2 e147 10.1371/journal.pone.0000147 17215958 PMC1764720

[B22] Busscher JF van Duijkeren E Sloet van Oldruitenborgh-Oosterbaan MM The prevalence of methicillin-resistant staphylococci in healthy horses in the Netherlands Vet Microbiol 2006 113 131 136 10.1016/j.vetmic.2005.10.028 16303264

[B23] Moodley A Guardabassi L Clonal spread of methicillin-resistant coagulase-negative staphylococci among horses, personnel and environmental sites at equine facilities Vet Microbiol 2009 137 397 401 10.1016/j.vetmic.2009.01.034 19251386

[B24] Corrente M D'Abramo M Latronico F Greco MF Bellacicco AL Greco G Martella V Buonavoglia D Methicillin-resistant coagulase negative staphylococci isolated from horses New Microbiol 2009 32 311 314 19845115

[B25] Gillespie BE Headrick SI Boonyayatra S Oliver SP Prevalence and persistence of coagulase-negative *Staphylococcus *species in three dairy research herds Vet Microbiol 2009 134 65 72 10.1016/j.vetmic.2008.09.007 18950962

[B26] Sawant AA Gillespie BE Oliver SP Antimicrobial susceptibility of coagulase-negative *Staphylococcus *species isolated from bovine milk Vet Microbiol 2009 134 73 81 10.1016/j.vetmic.2008.09.006 18950969

[B27] Rajala-Schultz PJ Smith KL Hogan JS Love BC Antimicrobial susceptibility of mastitis pathogens from first lactation and older cows Vet Microbiol 2004 102 33 42 10.1016/j.vetmic.2004.04.010 15288925

[B28] Kunz F Corti S Giezendanner N Stephan R Wittenbrink MM Zweifel C Antibiotikaresistenz von *Staphylococcus aureus *und Koagulase-negativen Staphylokokken isoliert aus Mastitismilchproben von Schafen und Ziegen Schweiz Arch Tierheilk 2010 153 63 69 10.1024/0036-7281/a000152 21274832

[B29] Virdis S Scarano C Cossu F Spanu V Spanu C De Santis EP Antibiotic Resistance in *Staphylococcus aureus *and Coagulase Negative Staphylococci Isolated from Goats with Subclinical Mastitis Vet Med Int 2010 2010 517060 20445785 10.4061/2010/517060 PMC2860459

[B30] Contreras A Luengo C Sanchez A Corrales JC The role of intramammary pathogens in dairy goats Livestock Production Science 2003 79 273 283 10.1016/S0301-6226(02)00172-0

[B31] Swenson JM Brasso WB Ferraro MJ Hardy DJ Knapp CC Lonsway D McAllister S Reller LB Sader HS Shortridge D Correlation of cefoxitin MICs with the presence of *mecA *in *Staphylococcus *spp J Clin Microbiol 2009 47 1902 1905 10.1128/JCM.02304-08 19357210 PMC2691132

[B32] Mehrotra M Wang G Johnson WM Multiplex PCR for detection of genes for *Staphylococcus aureus *enterotoxins, exfoliative toxins, toxic shock syndrome toxin 1, and methicillin resistance J Clin Microbiol 2000 38 1032 1035 10698991 10.1128/jcm.38.3.1032-1035.2000 PMC86330

[B33] Carbonnelle E Beretti JL Cottyn S Quesne G Berche P Nassif X Ferroni A Rapid identification of Staphylococci isolated in clinical microbiology laboratories by matrix-assisted laser desorption ionization-time of flight mass spectrometry J Clin Microbiol 2007 45 2156 2161 10.1128/JCM.02405-06 17507519 PMC1932985

[B34] Dubois D Leyssene D Chacornac JP Kostrzewa M Schmit PO Talon R Bonnet R Delmas J Identification of a variety of *Staphylococcus *species by matrix-assisted laser desorption ionization-time of flight mass spectrometry J Clin Microbiol 2010 48 941 945 10.1128/JCM.00413-09 20032251 PMC2832446

[B35] Poyart C Quesne G Boumaila C Trieu-Cuot P Rapid and accurate species-level identification of coagulase-negative staphylococci by using the *sodA *gene as a target J Clin Microbiol 2001 39 4296 4301 10.1128/JCM.39.12.4296-4301.2001 11724835 PMC88539

[B36] Zadoks RN Watts JL Species identification of coagulase-negative staphylococci: genotyping is superior to phenotyping Vet Microbiol 2009 134 20 28 10.1016/j.vetmic.2008.09.012 18930607

[B37] CLSI Performance standards for antimicrobial susceptibility testing; 18th informational supplement. CLSI document M100-S18. Clinical and Laboratory Standards Institute, Wayne, PA, USA 2008

